# Long-Term Follow-Up of Dogs and Cats after Stabilization of Thoracolumbar Instability Using 2-0 UniLock Implants

**DOI:** 10.1155/2022/5112274

**Published:** 2022-04-26

**Authors:** Julien Letesson, Bastien Goin, Jean Louis Trouillet, Paul Barthez

**Affiliations:** ^1^Lameilhé Veterinary Clinic, Castres, France; ^2^University of Lyon, VetAgro Sup, ICE, 69280 Marcy-l'Etoile, France; ^3^Univ Lyon, Université Claude Bernard Lyon 1, Univ Gustave Eiffel, IFSTTAR, LBMC UMRT 9406, F69622 Lyon, France; ^4^Novetech Surgery, 98000 Monaco, Monaco; ^5^VEDIM, 4979 Fingig, Luxembourg

## Abstract

Traumatic vertebral fracture or luxation often results in spinal instability requiring surgical stabilization. This study describes the long-term outcome of spinal stabilization using a unilateral 5-hole 2-0 UniLock implant in eight dogs and two cats with trauma-induced thoracolumbar vertebral luxation/subluxation and presumed instability, as assessed by a combination of preoperative radiographs and MRI using a 3-compartment method. The UniLock plate was secured with four monocortical locking screws in adjacent vertebral bodies. Additional pins and facet screws were used in several patients. Postoperative radiographs and MRI studies showed restoration of the main spinal axis in all patients and satisfactory implantation of the screws in the vertebral bodies, with no intrusion in the vertebral canal or in the adjacent intervertebral disc spaces. Neurological status improved in nine patients six weeks postoperatively. Partial implant failure was detected in three patients with no long-term consequences. After 12 months, seven patients reached full recovery with no neurological deficit, two patients were euthanized (including one owing to an unrelated condition), and one remained paraparetic. The results of this study demonstrate that using a 2-0 UniLock implant to stabilize the thoracolumbar spine results in satisfactory long-term recovery in most dogs and cats with traumatic spinal luxation/subluxation and presumed instability. Complications may occur but do not require revision surgery and do not affect clinical outcomes.

## 1. Introduction

Traumatic vertebral fracture or luxation often results in spinal instability requiring surgical stabilization [1–3]. In dogs and cats, these injuries are most commonly due to road traffic accidents (RTA) in 2/3 of the cases [4]. The T3-L3 spinal segment has been documented to be the most frequently affected in both dogs and cats, representing 48 to 58% of the injuries, followed by the lumbosacral (caudal to L3) segment and the cervical region [4, 5]. Dogs are more predisposed to subluxation (20% of cases) than cats (6%), which are more prone to fractures [5]. The decision to surgically stabilize the spine is based on clinical and neurological examinations and assessment of vertebral instability using diagnostic imaging. Although stress views are contraindicated in these patients, vertebral instability may be predicted on static images using the previously described 3-compartment method [6–8]. This method divides the vertebral unit into three parts: dorsal, intermediate, and ventral. Failure of at least two compartments is considered as an indication of vertebral instability. Although CT is currently considered as the gold standard for the evaluation of spinal osseous structures [6–8], MRI allows for accurate evaluation of the spinal cord, intervertebral disc (IVD), and surrounding soft tissue structures [7–16]. Radiographs have been shown to be less accurate than CT for the evaluation of patients with spinal trauma [6].

Historically, several methods of surgical spinal stabilization have been proposed [2, 3, 9, 17, 18], including the fluoroscopy-guided placement of external fixation [19, 20], the use of a tension band or lubra plates on the dorsal compartment [21–23], and the use of pins or screws inserted dorsally in the vertebral body and secured with polymethylmethacrylate (PMMA) [2, 9, 17, 24]. A recent study also reported the use of pedicle screws and PMMA to stabilize L6 vertebral body fractures [25]. Although PMMA is widely used to provide rigid and adaptable stabilization, it may be cumbersome [2, 3], make wound closure more difficult [2, 3, 9, 17], and introduce a large amount of foreign material in the paravertebral musculature. The exothermic reaction generated during polymerization may also cause damage to the surrounding tissues [1–3].

Safe corridors for pin or screw implantation in the thoracolumbar spine have been described in dogs [26] and cats [27] based on CT images, establishing safe insertion points and the optimal axis to maximize bone purchase [28, 29].

Although dorsal implantation has been shown to be feasible and safe in the lumbar region, it is impractical in the canine thoracic spine owing to vertebral conformation and the ribs.

A ventral approach after thoracotomy has been proposed for the thoracic spine [26, 30–33]. In the lumbar region, a lateral approach has been suggested to optimize bone purchase [29]. A biomechanical argument also encouraged such an approach: in an ex vivo L3-L4 model, a bilateral PMMA pin unit placed dorsally to the spine did not show any biomechanical superiority compared to a unilateral PMMA pin unit placed laterally to the vertebral body [34].

The use of plates anchored in the vertebral body has been considered recently with the advance of locking osteosynthesis implants [2, 3, 28, 35–38]. A nonlocking plate needs perfect contouring to achieve stability, generating friction at the bone-plate interface and high torque on the screws [3]. Plate contouring is not easy to achieve for the spine owing to the complex conformation of the bones. Locking plates may provide satisfactory anchoring with monocortical screws [2, 3, 36] without the need of being perfectly contoured [3]. They can be placed on a nontension surface of the bone [3, 30]. Locking plates have a low profile and provide closer to normal spinal rigidity than PMMA [3, 35, 36]. This is particularly important in areas where major flexion forces are present, that is, the thoracolumbar junction. Relative stability provided by a semirigid construct has been recommended to limit the risks of screw pullout and failure for ventral plating of the thoracolumbar junction [30].

The design and titanium composition of the 2-0 UniLock locking implants is appropriate for spinal stabilization and has the advantage of being more compatible with MR imaging [39]. They have lower stiffness than a standard stainless steel 316L implant [3, 40]. Moreover, titanium implants have shown advantageous yield stress compared to stainless steel on an ex vivo bovine spine model [41]. These implants were originally designed for maxillofacial surgery in humans [42]. They have already been used successfully in the management of cervical fracture-luxation and caudal cervical myelopathy in 2 mm and 2.4 mm diameters [28, 37, 38]. To our knowledge, the use of this implant to treat thoracolumbar instability in dogs and cats has not been reported.

The purpose of this study was to assess the short- and long-term outcomes of spinal stabilization using 2-0 UniLock implants in dogs and cats with trauma-induced vertebral instability, as assessed by a combination of radiographs and MRI.

## 2. Materials and Methods

Medical records of the Lameilhé Veterinary Clinic were examined. We selected patients who presented for thoracolumbar spinal trauma and were surgically treated with 2-0 UniLock implants between 2015 and 2020.

Inclusion criteria included radiographs and a preoperative MRI study showing vertebral subluxation and suspected instability based on the 3-compartment method, stabilization using 2-0 UniLock implants, and a minimal 12-month follow-up period. Patients with a corner endplate, spinous, and articular process fractures were included; patients with complete vertebral body fractures were excluded.

Species, breed, age, gender, and weight at the time of admission were collected from the medical records. The cause of trauma was recorded. All patients were secured on a radiolucent backboard and sedated with 0.2 mg/kg morphine (morphine chlorhydrate, Aguettant, France) before any additional procedure could be performed. Complete physical, neurological, and orthopedic examinations were performed in all patients. Neurological status was graded from 0 (paraplegia with no deep nociception) to 5 (spinal hyperesthesia only) using the previously published modified Frankel spinal cord injury scale [43]. Patients with associated traumatic lesions requiring immediate attention were treated as needed.

All patients had orthogonal (lateral and ventrodorsal) radiographs (flat panel detector, Ibis, Bergamo, Italy) of the thoracolumbar spine at the time of presentation. They were manipulated with caution to avoid further spinal damage. Radiographs were evaluated for signs of deviation of the main spinal axis, relative displacement of vertebrae, change in size of the vertebral canal, change in width of IVD spaces, and vertebral fractures.

All patients had an MRI study (VetMR, 0.18T, Esaote) upon presentation. MRI studies were performed within 48 hours of the injury in eight patients, within 72 hours in one patient, and within 15 days in another. All acquisitions were performed with patients in right lateral recumbency with a neutral position. The typical MRI acquisition protocol included sagittal and transverse spin-echo T1- and T2-weighted images followed by postcontrast sagittal and transverse spin-echo T1-weighted and dorsal GRE T1-weighted images taken after intravenous injection of 0.1 mmol/kg of gadoteric acid meglumine salt (Clariscan, GE, USA). In two patients, gadolinium was not used.

**Figure 1:**
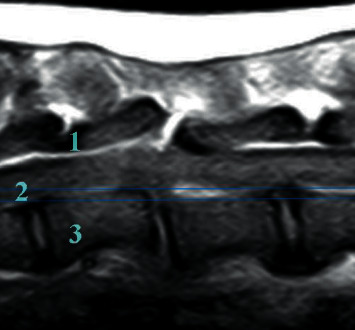
Sagittal T1-weighted MR image of the thoracic spine of one dog showing rupture of all three compartments, suggesting vertebral instability.

For each MRI study, the affected IVD space was recorded and assessed for size (collapsed, enlarged, and normal) and changes in T2-signal intensity. The dorsal and ventral aspects of the annulus fibrosus were assessed for signs of discontinuity. Fractures of adjacent vertebrae were also recorded. The vertebral canal was assessed for change in size, deviation of the main vertebral axis, presence of extradural material, and obliteration of the peridural fat. The spinal cord was assessed for changes in T2-signal intensity, diameter, and degree of deformation. Spinal cord deformation was defined as mild when the reduction of diameter was lower than 5%, moderate when it was between 5 and 20%, and severe for reductions exceeding 20%. Finally, epaxial muscles were evaluated for changes in T2-signal intensity and enhancement after gadolinium injection. The extent and side of the epaxial muscular changes were also recorded. Vertebral instability was assessed by combining results of radiographic and MRI studies, using the previously described 3-compartment method (Figure 1) [7]. The number of failing compartments was recorded for each patient.

Patients were prepared for surgery and anesthetized using the following protocol: premedication and analgesia were performed with 0.1 mg/kg/SC of morphine (morphine chlorhydrate, Aguettant, France) and 0.1 mg/kg IV of meloxicam (Metacam, Bohringer, France). Antibiotic prophylaxis consisted of 30 mg/kg/IV of cefazolin every two hours (Cefazolin, Panpharma, France). Induction was performed with 0.5 mg/kg/IV of diazepam (Valium, Roche, France) combined with 2 mg/kg/IV of alfaxalone (Alfaxan, Dechra, France). After intubation, anesthesia was maintained with oxygen and isoflurane (Isoflurin, Axience, France).

**Figure 2:**
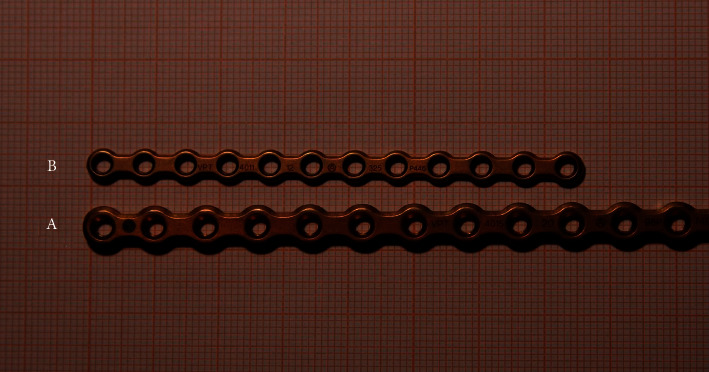
Two different 2-0 UniLock osteosynthesis plates used in the study. (A) 1.5 mm thick plates with an 8 mm interhole distance. (B) 1.3 mm thick plates with a 6.5 mm interhole distance.

Two versions of the UniLock 2-0 osteosynthesis plate (DepuySynthes, Switzerland) were used depending on the size of the vertebral bodies (Figure 2): 1.5 mm thick plates with an 8 mm interhole distance were used in seven patients, and 1.3 mm thick plates with a 6.5 mm interhole distance were used in three patients. The choice of the plate was made based on measurements of the vertebral body length on preoperative radiographs and sagittal MR images so that two locking screws could be placed in each adjacent vertebral body (four in total) with the central vacant hole located at the level of the affected IVD space. Plates were slightly contoured in seven patients to improve bone contact and decrease the risk of screw pullout. In one dog, one of the locking screws could not be placed satisfactorily and was replaced by a standard nonlocking screw (1/40 screw), as optimal placement of the locking screw could not be obtained owing to the locking guide's fixed angle.

**Figure 3:**
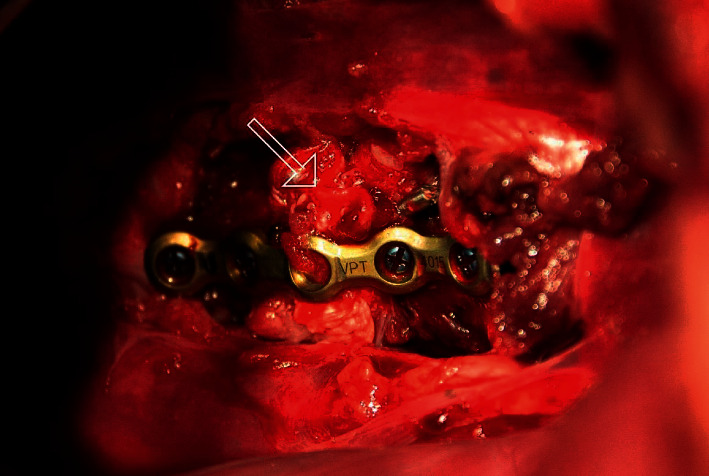
Surgical view after stabilization of Th11-Th12 IVD space of one dog with 2-0 UniLock osteosynthesis plate secured with four monocortical titanium locking screws and one pin via a thoracic approach. White arrow: complete loss of discal structure of Th11-Th12.

Patients with thoracic vertebral subluxation (four patients, all dogs) were positioned in right lateral recumbency. A left thoracotomy was performed at the level of the intercostal space corresponding to the site of the vertebral instability. Pulmonary expansion was controlled using positive pressure ventilation (Datex Aestiva/5, Datex Ohmeda, France). A Finochietto retractor and wet compresses were used to maintain the opening. In one dog, partial removal of the dorsal part of the 12th left rib was performed to widen the surgical field. The spine was approached ventrally to expose the vertebral bodies. Subluxation was reduced by manual traction of the vertebral bodies, and in three dogs, the alignment was maintained using a pin inserted caudocranially into both vertebral bodies. The precontoured 5-hole 2-0 UniLock plate was placed on the ventrolateral aspect of the vertebral bodies. It extended cranially and caudally to the following IVD space. It was held in place by a small pin inserted in the central hole. After securing the locking guide to the plate, only the cis-cortex was drilled with a 1.5 mm drill bit (DepuySynthes, Switzerland) in a laterodorsal direction before implanting the four locking monocortical screws (Figure 3).

**Figure 4:**
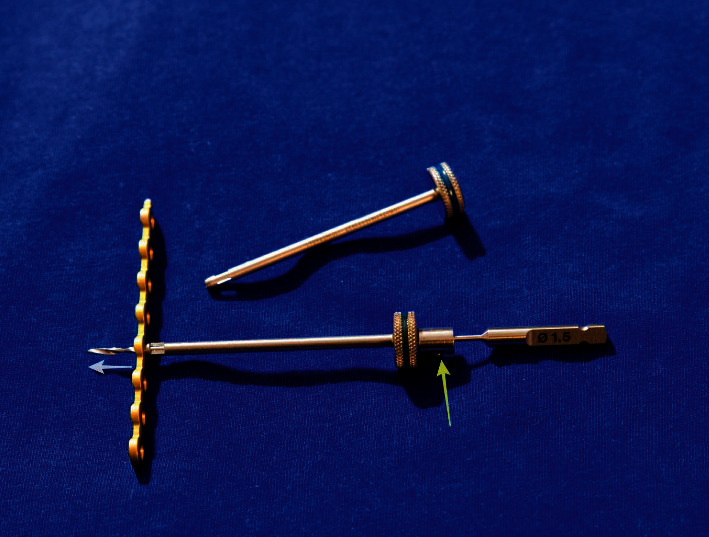
Photograph of plate and drill stop system showing drill stop mounted on 1.5 mm drill bit (yellow arrow) and predefined depth of drilling (white arrow).

A drill stop system (Figure 4) (Veterinary Instrumentation, Sheffield, UK) was used to help maximize bone purchase and remain monocortical. The depth of drilling was predefined using preoperative MRI images. The locking guide was used to block the drill at the planned depth (Figure 4). In one dog, extradural cord compressive material was removed using a foraminotomy with an additional lateral approach. Finally, a chest tube was inserted, and the intercostal space was closed conventionally plane by plane, associated with the restoration of the pleural vacuum.

**Figure 5:**
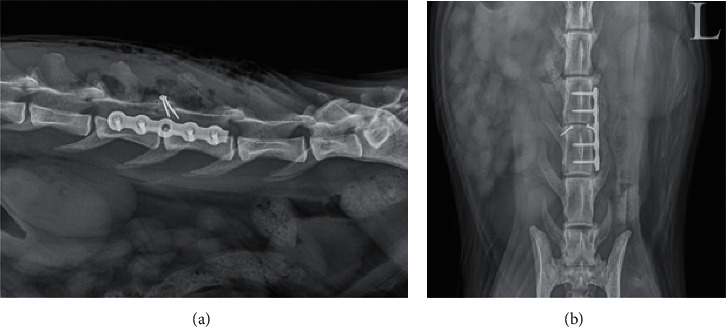
Postoperative lateral (a) and ventrodorsal (b) radiographs of lumbar spine of a cat suffering from L4-L5 injury showing 2-0 UniLock osteosynthesis plate secured with four monocortical titanium locking screws placed on the left side of vertebral bodies of L4 and L5 and two additional 1 mm stainless steel facet screws.

Patients with lumbar vertebral subluxation (six patients: four dogs and two cats) were positioned in ventral recumbency. A lateral approach to the lumbar spine was performed in three dogs. In the other three patients (two cats and one dog), a dorsal approach to the lumbar spine was performed to insert facet screws. The latter were used to maintain the reduction and alignment of the vertebral bodies during the placement of the plate. A single facet screw (2 mm UniLock nonlocking screw, Depuy Synthes, Switzerland) was used in one dog on the ipsilateral side of the bone plate. For the two cats, two 1 mm stainless steel screws (VOI, USA) were used, and a minimal extension of the dorsal approach was needed on the opposite side of plating (Figure 5). The subluxation was reduced by gentle manipulation of the vertebral bodies. The 2-0 UniLock osteosynthesis plate was then placed laterally between the articular and transverse processes of each vertebra. The screws were then implanted into the vertebral bodies. In dogs, the drilling entry point was at the level of the junction between the pedicle and the transverse process with a 60° angle between the sagittal plane and the direction of the drilling in a slightly ventral direction [26, 29]. In cats, the drilling entry point was at the base of the transverse process with an angle close to 90° to the sagittal plane (Figure 5) [27]. The depth of drilling was determined as described above, and a drill stop (Figure 4) was used to maximize the bone anchorage of the monocortical screws.

Orthogonal (lateral and ventrodorsal) radiographs were performed immediately after surgery in all patients to evaluate the reduction of the spinal subluxation, the restoration of the spinal axis, and the accurate placement of the implants. The position of the screws was assessed particularly to determine if they were monocortical.

A postoperative MRI study was performed in nine out of ten patients, using the same protocol as that of the preoperative study. MR images were evaluated for signs of paramagnetic artifacts produced by the implants, and, if present, their effect on the evaluation of the vertebral canal was assessed. The integrity of the vertebral canal and the size and shape of the spinal cord were evaluated for signs of persistent spinal cord compression.

After surgery, patients received 0.1 mg/kg SID of meloxicam (Metacam, Bohringer, France) orally for 15 days. A complete physical and neurological examination was performed after complete recovery from anesthesia. All patients were cage-rested for three weeks. A standardized postoperative physiotherapy program was performed, including standing sessions three times a day, mechanotherapy of the pelvic limbs, and gentle walking associated with abdominal support when voluntary movements were present [44].

A first follow-up evaluation was performed two to four weeks postoperatively in all patients. Additional follow-up evaluations were performed four to 28 weeks postoperatively in eight patients. They consisted in complete physical, neurological, and radiographic examinations. Long-term follow-up was performed 12 months postoperatively in eight surviving patients by telephone interview with the owner or referring veterinarian and a clinical exam or analysis of video clips of the patient's locomotion sent by the owner.

## 3. Results

Eight dogs and two cats met the inclusion criteria. Dogs were (mean ± SD) 6 ± 4.5 years old and weighted (mean ± SD) 16 ± 6 kg. There were six females and two males. Breeds included Border Collie (*n* = 2), Eurasier (*n* = 1), English Setter (*n* = 1), Cocker Spaniel (*n* = 1), Ratter (*n* = 1), Yorkshire Terrier (*n* = 1), and Fawn Brittany Basset (*n* = 1). The two cats were European Short Hair cats aged 1 and 2.5 years and weighing 3 and 5 kg, respectively. There were one male and one female. The cause of trauma was RTA in six patients. Other causes included a cow kick (*n* = 1), getting trapped in a gate (*n* = 1), a high-rise fall (*n* = 1), and hunting trauma (*n* = 1) (Table 1).

All patients had nonambulatory paralysis with signs of upper motor neuron (UMN) lesion in nine patients and signs of lower motoneuron (LMN) lesion in one cat with an L4-L5 lesion. Modified Frankel spinal cord injury grade was 3b in two patients (one dog and one cat), 2 in three patients, 1 in three patients, and 0 in two patients. One of the two patients with grade 0 was downgraded from 1 to 0 during the admission period (Table 1).

Crepitation on palpation of the spine was observed in all patients, suggesting vertebral fractures/subluxation, which were confirmed radiographically. All lesions were included in the T11-L5 segment, with three lesions at the T12-T13 level, three lesions at the L2-L3 level, and one lesion at T11-T12, L1-L2, L3-L4, and L4-L5 levels (Table 2). Based on lateral radiographs, deviation of the main spinal axis was identified in nine patients, with ventral displacement of the caudal spinal segment in seven patients (seven dogs) and dorsal displacement in two patients (two cats). Upon examination of the ventrodorsal radiographs, lateral deviation of the main spinal axis was observed in four patients. Endplate corner fractures were identified in two patients. Bilateral zygapophyseal joint luxation was observed in two patients (1 cat and 1 dog) (Table 2).

**Figure 6:**
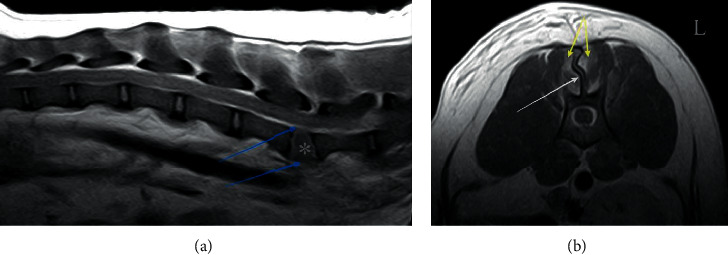
Postcontrast sagittal (a) and transverse (b) T1-weighted MR images of two different dogs showing rupture of dorsal and ventral annulus fibrosus of L3-L4 intervertebral disc (blue arrows), increase in the size of L3-L4 intervertebral disc space (^*∗*^), fracture of spinous process of L3 (white arrow), and enhancement of epaxial muscles (yellow arrows).

Based on MR images, the IVD space was collapsed in six patients and enlarged in four patients (Figure 6). There was a decrease in the T2-signal intensity of the IVD space in six patients and an increase in two patients. In these two patients, there was also an increase in the size of the IVD space. Rupture of the dorsal part of the annulus fibrosus of the affected disc was identified in all patients, and rupture of the ventral part of the annulus fibrosus was observed in six dogs (Figure 6). Subluxation or deviation of the main spinal axis was seen on MR images in seven patients (all dogs), with ventral displacement of the caudal segment in six dogs and right-sided displacement in one dog. Although they went undetected on radiographs, spinous process fractures were identified in three dogs (Figure 6), and articular process fractures were observed in two additional dogs (Table 2).

Extradural material resulting in obliteration of the epidural fat was observed in five patients. In one dog, this material was considered to cause spinal cord compression, justifying a foraminotomy to remove the compressing material in addition to spinal stabilization. An increase in T2-signal intensity of the spinal cord was identified in four patients. Deformation of the spinal cord was considered mild in two patients, moderate in four, and severe in one. In three additional patients, there was no evidence of spinal cord deformation. An increase in T2-signal intensity and enhancement of adjacent epaxial muscles was observed in seven patients (Figure 6). Changes were bilateral in six patients and unilateral in one. Muscle lesions extended between one and four vertebral bodies, mainly caudally to the injury.

Combining radiographic and MRI findings, instability was suspected in all patients (Figure 1), with all three compartments affected in six patients and two compartments affected in the remaining four (Table 3).

No patient experienced a worsening of their neurological status postoperatively. Based on postoperative radiographs, reduction of subluxation and restoration of the main spinal axis were identified in all patients (Figure 5). Implantation of the UniLock screws in the affected vertebral bodies was monocortical in 38 out of the 40 screws inserted (95%). In dogs treated with an intrathoracic surgical approach, the insertion of the pin bridging the affected intervertebral space was satisfactory in two out of three patients. In the remaining patient, the pin penetrated the vertebral endplate of T12, but it was not anchored in the body. No screws penetrated the adjacent healthy IVD space (Table 3).

**Figure 7:**
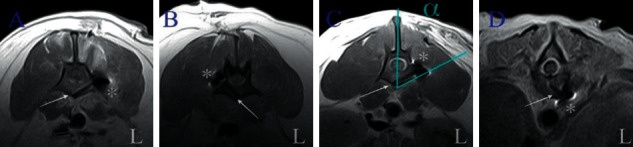
Postoperative transverse T1-weighted MR images of lumbar (a, b, and c) and thoracic (d) spine of four different dogs showing implantation of monocortical screws (arrow). Minor susceptibility artifacts are associated with orthopedic implants (^*∗*^). Angle (*α*) of implantation in lumbar vertebra is close to expected [26, 29].

Postoperative MRI studies showed an absence of spinal cord deformation in all nine patients on whom MRI was performed. No implant penetrated the vertebral canal (Figure 7). Minor susceptibility artifacts were produced by the stainless steel facet screw and additional pins used in the thoracic region, but no artifacts due to the UniLock 2-0 osteosynthesis plate were identified. Nevertheless, susceptibility artifacts did not interfere with the evaluation of the vertebral canal (Figure 7).

**Figure 8:**
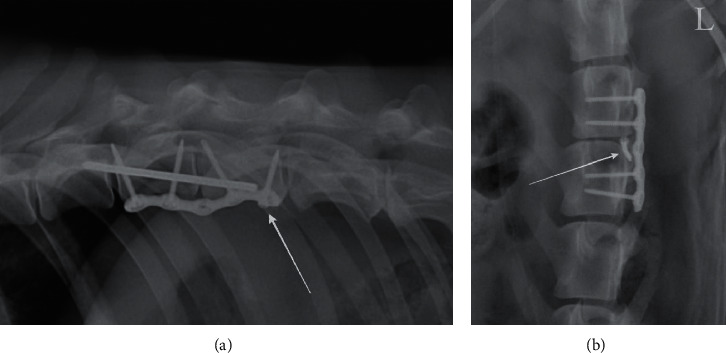
Lateral radiograph of the caudal thoracic spine (a) and ventrodorsal radiograph of the lumbar spine (b) three weeks postoperatively of two different dogs showing (a) slight withdrawal of most caudal screw (arrow) and (b) bending of facet screw (arrow).

At the three-week follow-up evaluation, neurological status had improved in eight patients with an improvement of 1 to 2 grades compared to the preoperative evaluation (Table 4). In the two remaining patients (one dog and one cat), no change in the neurological score was observed. These two patients were those with the worst neurological status upon admission (grade 0). The cat was euthanized three months postoperatively owing to poor quality of life. Radiographs performed during the first follow-up evaluation showed no evidence of recurrence of subluxation in any patient. Although no plate failure or displacement was identified, in two patients with a thoracic lesion (Table 3), two locking screws placed at the extremity of the plate slightly pulled out (Figure 8), and another screw broke at the junction between the shaft and the head. In another patient (one dog), one of the facet screws was bent but not broken, with no consequence on the plate (Figure 8). For these three patients with partial implant failure, radiographs performed during additional follow-up evaluations showed no further screw movements or implant failure. One dog died of an unrelated cause five months after surgery. In the remaining eight patients (seven dogs and one cat), neurological improvement continued during the follow-up period, including in the dog, which showed no improvement after three weeks.

After 12 months, remote evaluation of locomotion showed no gait abnormality nor signs of pain in seven patients. The remaining patient was able to maintain a standing position and perform a few voluntary movements but was still nonambulatory 15 months after the trauma.

## 4. Discussion

The current study examined thoracolumbar injuries, which are the most commonly injured area of the spinal column in cats and dogs [4, 5]. This provides homogeneity in the study population despite the small sample size (*n* = 10). We also focused on vertebral luxation/subluxation, excluding patients with vertebral body fractures, so that the locking implant could be fully secured in the adjacent vertebral bodies. Road traffic accident was the predominant cause of trauma in six out of ten cases, as previously reported [5]. The overrepresentation of dogs in this sample may be due to a difference in medicalization between dogs and cats in our referral population. Cats are also more prone to vertebral body fracture than to luxation/subluxation in case of spinal trauma [5].

A combination of radiographs and MRI was used in this study to assess pre- and postsurgical spinal changes. Although CT has been shown to be more sensitive than radiographs in the diagnosis of spinal cord trauma [6], this modality was not easily available during the course of this study. MRI has been reported to be an accurate method to assess vertebral instability resulting from traumatic injuries [7, 8]. In addition, the excellent contrast resolution of the soft tissues provided by MRI allows the IVD space, the extradural space, the meninges, the spinal cord, and the surrounding epaxial muscles to be assessed [7–14]. Combining CT to assess bony structures and MRI to assess soft tissue structures has been suggested for a thorough evaluation of the injured spine [8]. Radiography and MRI were considered to be complementary for the same reasons and, although not ideal, were used in this study for logistic reasons. Indeed, radiographic findings exclusively included changes in bone integrity (fractures) and relative position of the vertebrae (luxation; subluxation). Interestingly, ventral displacement of the caudal segment was observed only in dogs and dorsal displacement only in cats. This species difference was demonstrated previously in a larger cohort and may be due to anatomical differences and forces involved during trauma [5]. It has been pointed out that dorsal displacement of the caudal segment is almost impossible without a fracture of the articular processes or luxation of the zygapophyseal joint [5].

MRI studies confirmed bony changes in most cases, although the latter were often conspicuous on radiographs. However, MRI also revealed several undetected fractures, all located in the dorsal compartment. MRI studies provided invaluable information on the soft tissue structures of the spine and were also particularly helpful in assessing spinal cord instability using the three-compartment method [7]. The IVD is central in this evaluation because the annulus fibrosus of the disc is involved in both ventral and intermediate compartments [7]. The integrity of the dorsal and ventral annulus fibrosus on MR images was a key point in assessing spinal instability in our patients.

The T2-signal intensity of the affected IVD space was modified in most patients. As observed in six patients, a decrease in T2-signal intensity may have been the result of traumatic disc extrusion or previous disc degenerative changes. The increase in T2-signal intensity of the IVD space observed in two patients in association with an increase in the size of the IVD space may have been caused by fluid/blood accumulation due to local depression.

MRI studies were invaluable to identify the spinal cord changes, that is, deformation [10] and increase in T2-signal intensity [7, 11, 12], which were not visible on radiographs. Spinal cord deformation was mainly associated with changes in the main spinal axis due to luxation/subluxation and compression by extruded disc material (*n* = 1). No spinal cord deformation was observed in postoperative MRI studies, owing to the reduction of the subluxation/luxation and removal of the extruded disc material. The increase in T2-signal intensity of the spinal cord in patients with traumatic injury may have been caused by edema, hemorrhage, ischemia, or cell injury caused by direct spinal cord contusion or laceration [7]. Although our sample size did not allow us to perform valuable statistical analysis, all four patients with T2-hyperintense intramedullary changes were graded 0 or 1 upon admission, suggesting severe spinal cord injuries. Changes in signal intensity of the spinal cord are considered to be a valuable prognostic indicator in patients with spinal cord injury due to disc disease [11, 12, 16]. Nevertheless, three patients with initial intramedullary T2-signal changes had a satisfactory recovery after surgical stabilization, thus suggesting that these changes were potentially reversible.

Changes in T2-signal intensity of the epaxial muscles adjacent to the spinal injury were observed in most patients in the current study, corroborating previous similar findings [7]. These changes were thought to be primarily due to edema, inflammation, contusion, or laceration as a result of direct trauma. However, they could also be due to secondary muscle ischemia, spasm, or denervation, as previously described in patients with acute disc extrusion [13–15]. No change in signal intensity of the hypaxial muscles was observed in this study, supporting direct trauma as the most likely cause of epaxial muscles signal changes rather than secondary lesions.

The results of this study indicate that use of the 2-0 UniLock implant to stabilize the thoracolumbar spine results in satisfactory long-term recovery in most dogs and cats with traumatic spinal subluxation and presumed instability.

Using a titanium implant allows MRI examinations to be performed since titanium causes very few paramagnetic artifacts [3, 39], as confirmed by postoperative and follow-up studies. We used such a construct to provide relative stability to the spine [30]. The use of a VCP plate was previously recommended as an elastic stabilization of the spinal unit in order to limit the risks of screw pullout in a mobile part of the spine, that is, the thoracolumbar junction [30]. Using titanium, whose Young's modulus is only half that of 316L stainless steel [40] and closer to that of cortical bone, could thus be advantageous. A study using an ex vivo bovine spine model also suggested that titanium has a lower fatigue failure risk than a stainless steel construct thanks to greater yield stress [41].

However, the choice of this implant was primarily dictated by the intrinsic characteristics of the plate, allowing it to be centered on the affected IVD space and to insert two screws in each vertebral body without any risk of intrusion in the adjacent IVD space. Limiting the number of locking screws reduces the rigidity of a locking construct [3].

Plates of two different sizes were used, depending on the size of the vertebral bodies as measured on radiographic and sagittal MR images. Although none of our patients exceeded 25 kg, larger plates may be needed for larger dogs.

Locking implants have several advantages. They preserve reduction and [3] do not need to be placed on a tension bone surface. They can therefore be used on the vertebral body in the ventral compartment [3]. UniLock plates have also been found to have better resistance to cyclic loads when used with locking implants as opposed to standard ones in a human mandible model [42]. Perfect contouring of the plate to the bone surface is not needed with this type of implant [3], which is a major advantage in this region where the shape of the vertebrae is irregular. In the current study, although the plate was slightly contoured to ease bone-plate contact, it was not adapted to the bone surface perfectly, thus saving surgical time. The presence of a vacant hole at the level of the IVD space did not seem to weaken the fixation [3], as no plate breakage was observed in our series. This hole may even be used to center the plate on the injured intervertebral space during surgery. In our construct, this central vacant hole could also limit the stiffness of the fixation [3].

To anchor the UniLock plate, we preferred using monocortical locking screws. Their biomechanical performance has been shown to be equivalent to that of bicortical screws or pins implanted in the cervical spine in ex vivo models [18]. In a monocortical setting, the unique bone-screw interface is critical, and screw pullout may be a common complication [3]. To limit these risks, we slightly contoured the implant to obtain divergent screws [3]. We used a self-tapping locking implant with an advantageous thread-shaft ratio, and we used a drill stop with a preset drilling depth to optimize anchorage [28]. The main advantage of using monocortical locking screws is a lower risk of intruding into the vertebral canal and causing damage to the adjacent vascular structures. In our series, none of the screws penetrated the vertebral canal, and all screws except two were indeed monocortical. The risk of damage to the vascular structures is particularly high in the thoracic region, where the proximity of major vessels and the limited dorsal implantation corridor in dogs [26, 30–33] justify a ventral thoracotomy approach. A left thoracotomy should be preferred because of the position of the caudal vena cava [30, 32]. This ventral approach also allows for better visual control of the reduction of the luxation/subluxation and easier fixation of the implants in the vertebral bodies. A ventral approach is not justified in the lumbar region, where the dorsal implantation corridors are wider [26, 45] and underlying vascular structures, that is, aorta and caudal vena cava, are protected by the quadratus lumborum and psoas muscles. We opted for a lateral approach to the vertebral bodies in the lumbar region to have better exposure, limit muscle interference with the locking guides, limit further trauma to the epaxial muscles, and improve bone purchase as well as the angle of implantation [29].

A dorsal approach was performed only when a facet screw had to be placed. Epaxial muscles are directly responsible for the extension of the spine [13] and play an essential role in its stability [20]. Protecting these muscles was thus a major concern during surgery and contributed to choosing this approach and the use of a unilateral UniLock implant.

Partial implant failure occurred only in three dogs in our study and did not result in instability requiring revision surgery. These complications occurred with screws placed at the extremity of the plate in two patients with thoracic subluxation associated with articular process fractures. These patients had a major disc injury with complete rupture to the annulus fibrosus dorsally and ventrally, causing damage to all three compartments. The annulus fibrosus is a key element in the rotational stability of the spine, especially when the intervertebral facets are absent [46]. In both patients, we concluded that excessive rotational instability was the most likely cause of partial implant failure, despite the use of a pin securing the two vertebral bodies. An ex vivo study suggested that when a unilateral articular process fracture or luxation is present, a locking plate should be placed on the ipsilateral side to protect the construct, thus allowing the plate to act as a tension band during lateral bending [35]. We were unable to verify this hypothesis in our two patients. We placed both plates on the left side, and the articular process fracture was on the left in one patient and on the right side in the other patient.

We also observed the bending of a facet screw in the lumbar region without plate failure in one dog with suspected major instability (failure of all three compartments). In addition to rupture of the annulus fibrosus, this patient had major damage in the dorsal compartment (bilateral zygapophyseal joint luxation, fracture of the spinous process of L3, and marked hyperintensities of epaxial muscles). Facet implants were used to protect the plate from strain when it is not tensioned. According to Shores and Walker, ventral flexion forces are the most important ones to neutralize during spinal subluxation, especially when the dorsal compartment is involved in association with complete rupture of the annulus fibrosus [22, 45]. Experimental studies have shown that implants positioned in the ventral and dorsal compartments [22, 47] and in an orthogonal position [30] seem to better resist these bending forces. Facet screws also helped maintain the reduction and alignment of the vertebral bodies during the placement of the plate. Compressive material, when present, was therefore removed by performing a foraminotomy to preserve the articular processes and avoid any subsequent destabilization of the spine.

The small number of patients and the retrospective nature of this series are major limitations. We chose to select patients with the same kind of spinal injury (luxation/subluxation) in the same location (thoracolumbar area) and treated with the same kind of implants to have a homogeneous and meaningful sample. This study may be considered as a preliminary study justifying further research with larger samples of patients suffering from thoracolumbar luxation/subluxation and treated with a unilateral UniLock plate.

## 5. Conclusion

In this limited series, the relative stability provided by a 2-0 UniLock plate with monocortical locking screws suggests that it may be sufficient to limit the risk of implant failure and provide support for healing. The few minor surgical complications did not interfere with the clinical outcomes and did not require revision surgery. The major advantage of using a low-profile locking plate in patients with thoracolumbar instability is to limit both the surgical approach and the stiffness of the treated IVD space, subsequently reducing the surgical footprint.

## Figures and Tables

**Table 1:** Patient signalment, cause of trauma, and neurological status upon admission.

Species	Breed	Age (years)	Gender	Weight (kg)	Cause of trauma	Neurolocalization	Neurological status
Dog	Eurasier	2	M	23	RTA	UMN	Gr1 evolving to Gr 0
Dog	English Setter	3	F	18	RTA	UMN	Gr1
Dog	Cocker Spaniel	8	F	17	Trapped in gate	UMN	Gr2
Dog	Yorkshire	11	F	4	RTA	UMN	Gr2
Dog	Ratter	14	M	15	RTA	UMN	Gr3b
Dog	Border Collie	1.5	F	16	High-rise fall	UMN	Gr1
Dog	Fawn Brittany Basset	7	F	12.5	Hunting trauma	UMN	Gr2
Dog	Border Collie	3	F	20	Cow kick	UMN	Gr1
Cat	European Cat	1	F	3	RTA	UMN	Gr3b
Cat	European Cat	2.5	M	5	RTA	LMN	Gr0

RTA: road traffic accident; UMN: upper motor neuron; LMN: lower motor neuron.

**Table 2:** Preoperative radiographic and MRI findings.

Radiographic findings						MRI findings												
						Intervertebral disc space			Vertebrae	Vertebral canal		Spinal cord			Epaxial muscles			
Species	IVD space	Axial displ ML views	Lateral displ DV views	Caudal segment displ	Vertebral plateau fracture	Collapsed/enlarged/normal	T2 signal of the disc	Rupture of the annulus fibrosus	Fractures	Subluxation/change in vertebral canal diameter or main vertebral axis	Extradural material/obliteration peridural fat	Hypersignal T2	Compression	Hypersignal T2	Enhancement	Side	Extent (nb vertebral bodies)	Extent (cranial-caudal)
Dog	Th12-Th13	Yes	No	Ventral	No	Collapsed	Decreased	Dorsal and ventral	Spinous process fracture of Th13	Mild ventral displacement of main spinal axis caudal to Th13	Yes	No	Moderate	Yes	Yes	Bilateral, but more severe on the right side	4	Caudal (1 Cr + 3 Cd)
Dog	T11-T12	Yes	Yes	Ventral	No	Collapsed	Decreased	Dorsal and ventral	Right cranial articular process of T12	Slight ventral displacement of T12 relative to T11	Slight	Yes	Moderate	Yes	N/A	Both	1	Caudal
Dog	L3-L4	Yes	No	Ventral	No	Severely enlarged	Increased	Dorsal and ventral	Spinous process deformation of L3	Mild ventral displacement of main spinal axis caudal to L4	No	No	No	No	No	N/A	N/A	N/A
Dog	L2-L3	Yes	Yes	Ventral	No	Enlarged	Normal	Dorsal and ventral	No	Ventral displacement of main spinal axis caudal to L2-L3	No	No	No	No	No	N/A	N/A	N/A
Dog	Th12-Th13	No	Yes	No	No	Slightly collapsed	Decreased	Dorsal	No	No	Slight	No	Moderate	Yes	Yes	Left	1.5	Caudal
Dog	L2-L3	Yes	No	Ventral	Yes	Collapsed	Severely decreased	Dorsal	Spinous process fracture of L3	Rightward deviation of main spinal axis caudal to L2-L3	Slight	Yes	Mild	Yes	Yes	Both	2	Central
Dog	L1-L2	Yes	No	Ventral	No	Severely enlarged	Increased	Ventral and dorsal	No	Ventral displacement of main spinal axis caudal to L2	No	No (T2 quality)	Moderate	No	Yes	Both	1	Caudal
Dog	Th12-Th13	Yes	No	Ventral	No	Slightly enlarged	Normal	Ventral and dorsal	Left cranial articular process of T13 fracture	Mild ventral displacement of main spinal axis caudal to Th13	Yes	Yes	Severe	Yes	Yes	Both	1	Caudal
Cat	L4-L5	Yes	No	Dorsal	No	Slightly decreased	Severely decreased	Dorsal	No	No	No	Yes	No	Yes	Yes	Both	1.5	Caudal
Cat	L2-L3	Yes	Yes	Dorsal	Yes	Collapsed	Decreased	Dorsal	No	No	No	No	Mild	No	N/A	N/A	N/A	N/A

Displ: displacement; ML: mediolateral, DV: dorsoventral, Nb: number, N/A: nonapplicable, Cr: cranial, and Cd: caudal.

**Table 3:** Neurological status upon admission with spinal injury assessment, implants used, clinical outcome, and radiographic evolution of implants at 3 weeks postoperatively.

Species	Injured site	Neurological status before surgery	Number of injured compartments	Changes in T2-W sequences/deformation of spinal cord (no, mild, moderate, and severe) according to MRI cross-section measurement	Compressive material	Surgical approach	Main implant in all cases: 5-hole 2-0 UniLock plate	Radiographic evaluation at 3 weeks postop	Neurological status at 3 weeks postop
Dog	Th12-Th13	Gr0 MNC	3	No change in the spinal cord Moderate deformation	Discal material on the left side of cranial border of Th13	Thoracic, foraminotomy	4 locking screws, 1.5 mm pin Plate A	Implant in place No loss of vertebral alignment	Gr0
Dog	Th11-Th12	Gr1 MNC	3	Increased intensity of T2 signal in spinal cord Moderate deformation	None	Thoracic	4 locking screws, 1.5 mm pin Plate A	Pullout of distal locking screw No loss of vertebral alignment	Gr4
Dog	L3-L4	Gr2 MNC	3	No change in spinal cord signal No deformation	None	Lateral lumbar	4 locking screws, all monocortical Plate A	Implant in place No loss of vertebral alignment	Gr4
Dog	L2-L3	Gr2 MNC	2	No change in spinal cord signal No deformation	None	Lateral lumbar	1 cortical screw, 3 locking screws, and 2 bicortical screws Plate B	Implant in place No loss of vertebral alignment	Gr4
Dog	Th12-Th13	Gr3b MNC	2	No change in spinal cord signal Moderate deformation	None	Thoracic	4 locking screws Plate B	Implant in place No loss of vertebral alignment	Gr4
Dog	L2-L3	Gr1 MNC	3	Increased intensity of T2 signal in spinal cord Mild deformation	None	Lumbar	4 locking screws, all monocortical Plate A One 2 mm UniLock facet screw	Bending of facet screw No loss of vertebral alignment	Gr3b
Dog	L1-L2	Gr 2 MNC	2	No change in spinal cord signal Moderate deformation	None	Lateral lumbar	4 locking screws, all monocortical Plate A	Implant in place No loss of vertebral alignment	Gr3a
Dog	Th12-Th13	Gr1 MNC	3	Increased intensity of T2 signal in spinal cord Severe deformation	None	Thoracic	4 monocortical locking screws Plate A 1.8 mm pin	Breakage of one distal locking screw and pullout of another at the end of plate No loss of vertebral alignment	Gr3a
Cat	L4-L5	Gr0 MNP	3	Increased intensity of T2 signal in spinal cord Mild swelling of spinal cord No deformation	None	Dorsal lumbar	4 monocortical locking screws Plate A Two 1 mm facet screws	Implant in place No loss of vertebral alignment	Gr0
cat	L2-L3	Gr3b MNC	2	No change in spinal cord signal Mild deformation	None	Dorsal lumbar	4 locking screws, 1 mm facet screw Plate B	Implant in place No loss of vertebral alignment	Gr4

UMN: upper motor neuron, LMN: lower motor neuron, Plate A: 1.5 mm thick plate with 8 mm interhole distance, and Plate B: 1.3 mm thick plate with 6.5 mm interhole distance.

**Table 4:** Follow-up of clinical cases.

Species	Preoperative	Immediate postoperative follow-up	Follow-up 1			Follow-up 2			Follow-up 3			Follow-up 4			Interview or follow-up >12 months
	MFS	MFS	NWAS	MFS	Type of follow-up	NWAS	MFS	Type of follow-up	NWAS	MFS	Type of follow-up	NWAS	MFS	Type of follow-up	
Dog	0 UMN	0 UMN	2	0	Radiographs	6	1	Radiographs							✔
Dog	1 UMN	1 UMN	3	4	Radiographs										✔
Dog	2 UMN	2 UMN	3	4	Radiographs	6	5	Radiographs							✔
Dog	2 UMN	2 UMN	3	4	Radiographs	6	4	Radiographs	9	5	Radiographs	16	NoNS	Radiographs	✔
Dog	3b UMN	3b UMN	3	4	Radiographs	6	5	Clinical exam							Death 5 months after trauma
Dog	1 UMN	1 UMN	2	3b	Radiographs	4	4	Radiographs							✔
Dog	2 UMN	2 UMN	4	3a	Radiographs	8	4	Radiographs	28	NoNS	Radiographs				✔
Dog	1 UMN	1 UMN	4	3a	Radiographs	18	NoNS	Radiographs							✔
Cat	0 LMN	0 LMN	3	0	Radiographs	15	0	Radiographs							Euthanasia 3 months after trauma
Cat	3b UMN	3b UMN	3	4	Radiographs										✔

MFS: modified Frankel score according to Levine et al. 2006 [43], NWAS: number of weeks after surgery, UMN: upper motor neuron, LMN: lower motor neuron, and NoNS: no neurological sign.

## Data Availability

All the data are presented in tables or figures directly in this paper.
